# Public perceptions of drinking water: a postal survey of residents with private water supplies

**DOI:** 10.1186/1471-2458-6-94

**Published:** 2006-04-11

**Authors:** Andria Q Jones, Catherine E Dewey, Kathryn Doré, Shannon E Majowicz, Scott A McEwen, Waltner-Toews David, Mathews Eric, Deborah J Carr, Spencer J Henson

**Affiliations:** 1 Division of Community Health, Faculty of Medicine, Memorial University of Newfoundland, St. John's, Newfoundland, A1B 3V6, Canada; 2 Department of Population Medicine, University of Guelph, 50 Stone Road West, Guelph, Ontario, N1G 2W1, Canada; 3 Foodborne, Waterborne and Zoonotic Infections Division, Public Health Agency of Canada, 160 Research Lane, Suite 206 Guelph, Ontario, N1G 5B2, Canada; 4 City of Hamilton Public Health and Community Services, 1 Hughson Street North, Hamilton, Ontario, L8R 3L5, Canada; 5 Department of Agricultural Economics and Business, University of Guelph, 50 Stone Road West, Guelph, Ontario, N1G 2W1, Canada

## Abstract

**Background:**

In Canada, the legal responsibility for the condition of private water supplies, including private wells and cisterns, rests with their owners. However, there are reports that Canadians test these water supplies intermittently and that treatment of such water is uncommon. An estimated 45% of all waterborne outbreaks in Canada involve non-municipal systems. An understanding of the perceptions and needs of Canadians served by private water supplies is essential, as it would enable public health professionals to better target public education and drinking water policy. The purpose of this study was to investigate the public perceptions of private water supplies in the City of Hamilton, Ontario (Canada), with the intent of informing public education and outreach strategies within the population.

**Methods:**

A cross-sectional postal survey of 246 residences with private water supplies was conducted in May 2004. Questions pertained to the perceptions of water quality and alternative water sources, water testing behaviours and the self-identified need for further information.

**Results:**

Private wells, cisterns or both, were the source of household water for 71%, 16% and 13% of respondents, respectively. Although respondents rated their water quality highly, 80% also had concerns with its safety. The most common concerns pertained to bacterial and chemical contamination of their water supply and its potential negative effect on health. Approximately 56% and 61% of respondents used in-home treatment devices and bottled water within their homes, respectively, mainly due to perceived improvements in the safety and aesthetic qualities compared to regular tap water. Testing of private water supplies was performed infrequently: 8% of respondents tested at a frequency that meets current provincial guidelines. Two-thirds of respondents wanted more information on various topics related to private water supplies. Flyers and newspapers were the two media reported most likely to be used.

**Conclusion:**

Although respondents rated their water quality highly, the majority had concerns regarding the water from their private supply, and the use of bottled water and water treatment devices was extensive. The results of this study suggest important lines of inquiry and provide support and input for public education programs, particularly those related to private water testing, in this population.

## Background

Over four million Canadians receive their drinking water from private water supplies, predominantly from groundwater wells [1]. In Canada, the legal responsibility for the condition of private water supplies, such as private wells and cisterns, lies with their owners [2]. There are reports, however, that Canadians with private water supplies test their water intermittently, if at all [1,3], and that water treatment within their homes is uncommon [1,3,4]. Similar situations have been reported in other developed countries [5-7]. Canadian private water supplies may pose a risk to public health; numerous studies report such water supplies in excess of the minimal acceptable standards for microbial and chemical contamination [1,3,4,8-10], and an estimated 45% of all waterborne disease epidemics in Canada involve non-municipal systems, largely in rural or remote areas [1].

Thus, it is especially important to understand the perceptions, needs and concerns of Canadians served by private water systems. Several surveys of water consumption behaviour and perceptions of alternative water sources, such as bottled water and water treated with in-home treatment devices, have been performed in North America [11-16]. However, these studies focused mainly on residents receiving municipally supplied water. To date, there have been no published studies investigating the perceptions held by residents served by private water supplies. Such understanding will enable public health professionals to better target public education and outreach activities, as well as address the needs and concerns of residents with private water supplies in their jurisdictions. This knowledge will also help to inform discussions of drinking water policy relating to private water supplies.

The purpose of this study was to investigate the public perceptions of water from private water supplies in the City of Hamilton, Ontario (Canada), with the intent of informing public education and outreach strategies within that population. The City of Hamilton has a population of approximately 500,000 and is a large, urban centre surrounded by suburban and rural areas. Approximately 20% of its households are served by private water supplies. Data reported include residents' perceptions of their private water supplies and alternative water sources, as well as their water testing behaviour and self-identified need for information.

## Methods

### Study design and sample selection

A cross-sectional postal survey of 246 residences classified as having private water supplies in the City of Hamilton, Ontario (Canada), was conducted in May 2004. Residential addresses were obtained from the Ontario Assessment System (OASYS) database, provided by the Municipal Property Assessment Corporation (MPAC). MPAC uses occupant information to create municipal and school board voter lists, juror lists, and population counts for each municipality in Ontario. To enable sample selection, we identified residences served by private water systems, including private wells and cisterns. Specifically, all residential addresses were mapped using postal codes and were overlaid on a digitized map of municipal water system distribution areas within a Geographic Information System (GeoMedia 5.0, Intergraph Corporation). The latter map was constructed using service data provided by the City of Hamilton's water treatment utilities. Residences not falling within municipal water system distribution polygons were classified as having private water sources and were included in the sampling frame.

Five hundred and fifty residences were selected using a random number generator, with 50 residences selected for the pilot of this study and 500 selected for the main study. In a similar study investigating the perceptions of water from municipal water supplies in this population (Jones et al., unpublished), two respondents (0.4%) informed us that we had incorrectly classified them as having municipal water. These respondents had private water supplies and were interested in participating in the private water study. Since all residences, for both studies, originally came from the same sampling frame (the OASYS database) and were randomly sampled using identical methods, on the same calendar date, we mailed to these two residences a private water survey package and included them in the current study. Therefore, 502 survey packages were distributed in total.

To assess the potential for selection bias, the demographic profiles of respondents and residents of the City of Hamilton were compared, using Statistics Canada 2001 census population data for the City of Hamilton. Proportions were tested with an overall goodness-of-fit test employing Chi-square analysis and Fisher's exact p-values; the latter were estimated using Monte Carlo simulation with 100,000 repetitions [17]. Where the overall test was significant, the observed and expected values in the subcategories were compared using Chi-square analyses to determine where the overall multinomial test failed to hold.

### Questionnaire development

The mail questionnaire was designed using information from the questionnaire design literature [18-24] and three focus groups conducted with residents on private water systems in the City of Hamilton [25]. The data generated from the focus groups informed the content and vocabulary of the questionnaire, as well as question categories and answer choices. The questionnaire used both open-ended and closed-ended questions; the latter included checklists and Likert scales. Five different five-point Likert scales were used, specifically to rate water quality (1 = very good and 5 = very poor) and to indicate the respondents' level of concern (1 = very concerned and 5 = very unconcerned), importance (1 = very important and 5 = very unimportant), assurance (1 = very sure and 5 = very unsure) and likelihood of use of media (1 = very likely and 5 = very unlikely).

The questionnaire was pre-tested with a convenience sample of individuals who received their household water from private systems. Questions that were unclear or otherwise problematic were revised. Subsequently, we performed a pilot study using 50 residences randomly selected from our sampling frame. Based on the results, minor revisions were made to decrease the length of the questionnaire.

### Response rate and survey methodology

To maximize our response rate, we used a number of methods [22], including a pre-notification letter, detailed cover letter, 8 × 11" survey booklet, a stamped, addressed return envelope, first-class postage and personal signing of all correspondence. Respondents were also given the opportunity to be eligible for one of three draws for $250 (CAD) upon returning the completed survey prior to the deadline. This deadline was initially set for two weeks after the mailing of the survey package, but was later extended by four weeks. Ten days after mailing the survey package, we sent a thank-you/reminder letter to all households; approximately 2 weeks after this, we sent a second letter to all non-responding households to encourage response. Six weeks after the initial mailing, telephone calls were made to non-responders, where possible, to encourage participation.

In all correspondence, we asked households to inform us if we had incorrectly classified their home as receiving water from a private supply either by contacting us directly (by email, phone or letter-mail) or returning the questionnaire blank. The number of misclassified residences was recorded for use in response rate calculations. The University of Guelph's Research Ethics Board approved the study.

### Analyses

The data were entered using the form-version entry function in Microsoft^® ^Access 2000 (Microsoft Corporation) and were validated prior to analyses. Specifically, the numeric database entries were checked against the originals for all of the returned questionnaires. Fields allowing string entries or involving skip patterns were examined for implausible values. In the few instances where these were encountered, the fields were relabeled as missing. Frequency distributions were calculated in STATA version 8.2 (StataCorp).

## Results

### Response rate

Of the 502 questionnaires mailed, 29 were returned to sender due to an invalid mailing address or lack of receipt of the package, and another 23 were returned because we had misclassified the household water system as private when it was actually municipal. These 52 residences were ineligible to participate and were thus excluded from the calculation of the response rate. In total, 246 questionnaires were returned completed, yielding an overall response rate of 54.7% (246/450). Not all questions were fully answered by all respondents; hence, some analyses were conducted with smaller sample sizes, as noted. The demographic profiles of respondents differed from the overall population of the City of Hamilton in some categories of age, household income and education (Table 1).

### Types of household water sources

Approximately 71% of households (168/238) were supplied by private water wells, 16% (39/238) by water cisterns and 13% (31/238) by both. Respondents with wells reported two types of well construction; 78.3% of wells (155/198) were drilled and 12.1% (24/198) were dug. Approximately 10% of respondents with wells (19/198) did not know what type of well they had. The depths of the wells were highly varied; approximately 13% were classified as shallow (less than 30 feet), 52% were moderate (30–89 feet) and 19% were deep (90 feet or deeper). Sixteen percent of respondents (31/196) did not know the depth of their well. Ninety-percent (179/198) of households owned the well that supplied water to their homes, while the remainder rented their homes, and hence the well, from the property-owner.

The use of multiple sources of cistern water was common; 38% (26/68) and 3% (2/68) of households with cisterns used two and three different sources of water respectively. Approximately 60% (41/68) and 9% (6/68) of households that used cisterns got their cistern-water from eaves troughs and private wells, respectively. Water haulage from external sources was also reported, with 62% (42/68) and 13% (9/68) of cisterns being filled with water from municipal water and other private water sources, respectively.

### Most common source of drinking water in the home

Water directly from the private water supply was the most common source of drinking water in the home for 33.5% (79/236) respondents. Approximately 35% (83/236) and 31% (74/236) of respondents reported bottled water and treated water from the private water supply to be the most common source of drinking water in the home, respectively.

### Perceptions of the quality of drinking water from private water supplies

Respondents were asked to judge the quality of the water from their private water supply, based on five sensory characteristics of drinking water. Many respondents rated the taste (40.7%; 96/236), smell (44.9%; 105/234), colour (51.5%; 121/235), clarity (48.3%; 113/234) and safety (38.2%; 87/228) of the water from their private supply as being "very good". Similarly, many respondents rated the taste (37.3%; 88/236), smell (36.3%; 85/234), colour (36.2%; 85/235), clarity (37.2%; 87/234) and safety (39.9%; 91/228) of the water from their private as being "good". Further, 27% (64/237) and 33.8% (80/237) of respondents were "very sure" or "sure" that the water from their private supply was safe for consumption, respectively.

However, 80.0% of respondents (188/238) were "very concerned" (111/238) or "concerned" (77/238), about the overall safety of the water from their private supply. About two-thirds of these respondents (123/188) explained their level of concern in response to an open-ended question, which is described by category in Table 2. The most common explanation was possible contamination of their private water supply; some were concerned with contamination in general, while others specified that their concerns involved pesticides/other chemicals or bacteria. Approximately 6% of respondents (15/238) were "unconcerned" or "very unconcerned" with the overall safety of the water from their private supply. Explanations were provided by 73% (11/15) of these respondents. Fifty-eight percent (7/11) were unconcerned because tests of their water had been okay in the past and they had good well management practices. A third (4/11) were unconcerned because they used water treatment devices. One respondent was unconcerned because they did not drink the water from their private supply.

Respondents also indicated their level of concern regarding the possible presence of various contaminants in their water supply (Table 3). Most respondents had concerns regarding bacterial contamination; approximately 62% and 28% of respondents reported being "very concerned" and "concerned", respectively.

### Water treatment devices: use and perceptions

**Figure 1:**
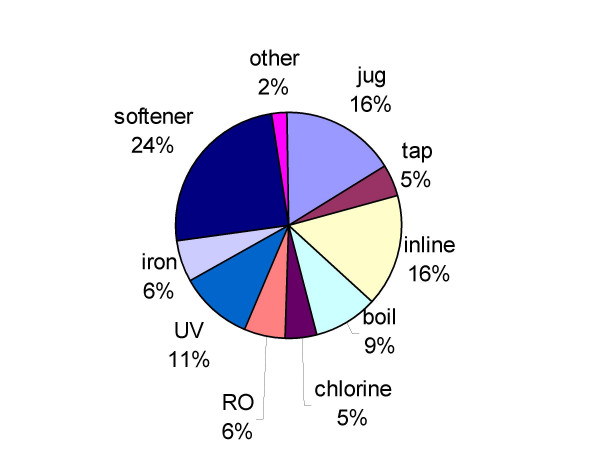
Frequency distribution of 266 treatment devices used to treat private water supplies (City of Hamilton, Ontario, 2004) (n = 130 households)* Jug = jug filter (e.g. Brita filter) Tap = tap filter Inline = inline filter Boil = boiling Chlorine = chlorine added RO = reverse osmosis UV = ultraviolet light Iron = iron removal device Softener = water softener Other = other device * Total number of devices exceeds total number of respondents because of multiple devices per respondent

Approximately 56% of respondents (130/234) treated the water from their private water supply for drinking purposes. The types of devices reported were varied (Figure 1). The use of multiple devices within homes was common; 36 (28%), 22 (17%) and 19 (15%) households used two, three or four devices, respectively. A water softener was common to all combinations of treatment devices. Table 4 summarizes the factors that were "very important" and "important" in the respondents' decision to treat their water for drinking purposes.

### Bottled water: use and perceptions

Approximately 61% of respondents (148/243) reported drinking bottled water in their home instead of the water from their private supply. The factors that were "very important" and "important" in respondents' decision to drink bottled water in the home are listed (Table 4).

### Public education

The desire for more information pertaining to private water supplies was common. Approximately 47% (111/237) and 34% (80/237) of respondents said it was "very important" or "important", respectively, that they receive more information pertaining to water testing. Specifically, information on where they could have their water tested, how often it should be tested and what tests should be performed was desired. Approximately 35% (79/226) and 30% (68/226) of respondents said it was "very important" or "important", respectively, that they be able to learn the test results from other private wells in their neighbourhoods, providing that the owner's name and address could be kept confidential. Finally, 34.4% (78/227) and 36.6% (83/227) of respondents said it was "very important" or "important", respectively, that they receive advice on water treatment options.

Respondents indicated how likely they would be to use various media to obtain information pertaining to private water supplies (Table 5). Nearly 85% (192/227) of respondents reported being "very likely"/"likely" to read a flyer or brochure mailed to their homes. The second medium most likely to be used was the newspaper.

### Water testing

Approximately 21% of households (51/242) had never tested the water from their private water supply. Of those who had tested their water (191/242), 88% had tested for *E. coli *and total coliforms. Approximately 72% of respondents (173/240) were aware that testing of water for *E. coli *and total coliforms was provided at no charge within the City of Hamilton. Testing for other parameters, including other bacteria (20%; 37/186), heavy metals (23.7%; 44/186), nitrates (22.6%; 42/186), pesticides (23.1%; 43/186) and sodium (16.7%; 31/186) was uncommon. Further, 20% of households (37/186) who had tested their water did not know what tests had been done.

The frequency with which households tested their water is summarized (Table 6). In response to an open-ended question, respondents explained why they did not test their private water supply or test it more frequently. The categorized responses are shown (Table 7) with the most common explanation relating to the inconvenience of the testing process. Table 8 summarizes the proportion of respondents that thought various methods would help to increase the frequency of water testing among households with private water supplies in the City of Hamilton.

## Discussion

Most respondents rated the quality of their private water supplies highly and 60% were sure it was safe for consumption. However, 80% of respondents reported having at least some concerns with the quality of their water. A possible explanation for this apparent discrepancy may be related to a perceived lack of health problems. Only four percent of respondents suspected that they or a family member had become ill as a result of a private water supply in Hamilton or Canada (data not shown). Thus, if there is no obvious evidence of illness or negative effects from their water, residents may deem it safe for consumption, yet still have concerns regarding its quality.

The most common concern reported was contamination of private water supplies, with either microbiological pathogens or chemicals. This was also indirectly illustrated in the respondents' concerns with the waterborne outbreak of *E. coli *in Walkerton, Ontario in 2000 [26], and the susceptibility of their water aquifers to external factors like construction and building development. Previous studies have indeed shown contamination of Canadian private water supplies with *E. coli*, nitrates and sulphates [3,4,8,9,27]. The participants highlighted the importance of a good quality water supply and the fundamental necessity of water and the effect water can have on health. Similarly, 60% of respondents in a U.S. survey of municipal water quality said that the quality of drinking water affects their health and 50% were concerned about possible health-related contaminants in the water supply [28]. Drinking water appears to be an important issue to the North American public.

Not all respondents, however, were concerned with the quality of water from their private supplies. Unfortunately, the grounds on which respondents based their lack of concern may or may not be well founded. For example, the most common explanation for participants' confidence was that previous water test results had been acceptable. However, few households tested their water regularly. Further, because water contamination can be intermittent and dependent on numerous factors, including well construction and weather [29], the main factor used in respondents' decision-making regarding the safety of their water might not be appropriate. Other respondents were not concerned with their water quality because they used water treatment systems. However, while it is recommended that the devices be certified with the National Sanitation Foundation, there is currently no legislation for water treatment devices in Canada [30]. Further, many manufacturers state their treatment devices to be suitable only for municipally treated or microbiologically safe water, which may be of limited use to residents with private water supplies [1]. Similarly, home treatment systems are not always effective in pathogen removal, particularly if they are not properly maintained [6,29,31]. In a survey of the microbiologic quality of private water supplies in England, several water samples tested positive for *E. coli *despite the use of treatment devices [6]. This further supports the finding that some residents are making decisions regarding their water safety based on inappropriate information.

Approximately one-third of respondents reported the most common source of drinking water in the home to be water treated with in-home devices. This is much higher than that observed in a Quebec study of private water supplies, where only 9% of 222 households drank treated water and 69% drank directly from the water supply [4]. The use of alternative water in the current study was also slightly higher than reported in other North American studies involving municipal water supplies [11-16]. The differences may relate to differences in the study periods, populations or water supply sources.

Perceived improvements in the aesthetic quality and safety of water were important reasons for the use of water treatment devices. Nearly half of the respondents reported using these devices to improve the aesthetics of the water from their private supplies. The majority of respondents however, used water treatment devices because of a perceived reduction in the concentration of bacterial (75%; 86/115), metal (59%; 67/115) and chemical (59%; 67/115) contaminants compared to the water directly from their private supply. Other studies report similar reasons for treatment device use; however, safety and health concerns of the water were emphasized less and the improvement of the aesthetic qualities of the water were emphasized more, compared to the current study [4,14].

Use of a variety of treatment devices was reported, with many households using more than one device for drinking purposes. In past studies of households with municipal water systems, the main types of devices reported employed activated carbon filtration, such as jug and tap filters [12,13,16]. In this study, devices tended to be more sophisticated, with water softeners, inline filters and ultraviolet light devices being the most common devices reported. While our questions asked specifically about the use of in-home treatment devices for drinking water, we suspect that many respondents included the use of devices for other purposes because the use of softened water for consumption tends to be uncommon. Hence, these results may overestimate the use of softeners for drinking purposes. Similarly, since our question omitted treatment devices for non-drinking purposes, the prevalence of use of treatment devices for all purposes in this population may be higher than that observed here.

Bottled water use in the home was also common. A perceived improvement in taste and the perception that bottled water had higher safety controls and reduced bacterial contamination, compared to the respondents' private water supply, were important reasons for its use. A previous study in the United States reports reasons for bottled water use to include convenience and preference over other beverages while outside of the home [15]. To improve comparison of bottled water to water from private household water supplies, we restricted the questions regarding bottled water to within-home use only, reducing the likelihood that convenience was included as a contributing factor for its use. The choice to use alternative water sources in the home therefore affirms that participants had concerns with the quality of the water from their private supplies.

Current provincial guidelines recommend testing private water supplies for indicator bacteria at least three times yearly [2]. Only 8% of households in this study tested the water from their private supply with a frequency that meets these guidelines. Approximately 21% of households never tested their water and 40% tested their water every two years or less often. This is comparable with results from other Canadian studies, which state that rural residents test their private water supplies intermittently, if at all [1,3]. The reasons for infrequent testing in these latter studies, however, were not determined. Given that monitoring of private water supplies is the sole responsibility of the owners of those supplies, it is important to increase the awareness and frequency of water testing in this population. An understanding of the reasons for infrequent testing is useful in this regard, and this study highlighted several. The main reason related to the inconvenience of having to travel into the city to pick-up and drop-off water collection bottles. Similarly, time constraints limited testing frequency because drop-off locations for water samples were open only during regular business hours and some participants reported not wanting, or being able to, take time off work to submit a water sample. Many respondents did not test their water supplies more frequently because there were no apparent health problems or noticeable changes to the water from their supply. Some waterborne illnesses however, are self-limiting or require chronic exposure. Further, some contaminants are odourless, colourless and tasteless [29], thus, waterborne hazards may be present without the owner's recognition. Members of a household may develop immunity to some infectious agents in their water, but visitors to the household may be susceptible and may, therefore, be at higher risk for illness [9,32]. Similarly, contamination of a private well can impact other nearby household water supplies via contamination of the aquifer [32]. Further, some respondents did not test their water regularly because they did not consume water from it. While this does reduce the risk of waterborne illness from this source, depending on the hazard involved, the risk might not be eliminated, as other routes of exposure like absorption or inhalation during bathing may be possible [33,34]. Hence, residents with private water sources could benefit from education initiatives that relay the benefits and importance of regular water testing.

There was also a tendency for past test results to dictate future testing behaviour; some households did not test their water because results in the past had been acceptable. However, the nature of private water supplies can vary greatly over time, and one test per year or less is likely to be insufficient in depicting the true nature of water quality. Further explanations for infrequent testing included procrastination and lack of information regarding water testing, including where and when samples could be tested. Approximately 28% of respondents were not aware that bacterial testing of water for *E. coli *and total coliforms was provided at no cost within the City of Hamilton. These explanations suggest the need to greater emphasize the importance of water testing and to increase the dissemination of water testing details in this population. A need to enhance the convenience of the process is also evident. Possible methods could include increasing the number of collection pick-up and drop-off locations and extending the hours for sample submission. While labour-intensive, given our results, it seems likely that these methods would increase the testing of private water supplies in this population. Ideally, suitable funding would be made available to the appropriate health departments to implement these measures. Community involvement or volunteer programs might also be an effective way to increase water sample collection and the distribution of collection bottles. Further, community centres, town halls or other convenient locations, might serve as water sample drop-off locations, where residents could deliver samples on specific dates and times.

While the majority of households had tested their private water supply, albeit infrequently, the testing was limited mainly to *E. coli *and total coliforms. While not assessed in this study, we suspect this may be related to the relative unavailability and the costs associated with the tests for other water parameters [2]. Some Canadian private water supplies have exceeded the guidelines for numerous hazards, including *Salmonella*, sulphates and nitrates, in addition to *E. coli *[1,3,4,8-10], therefore infrequent testing of water supplies may pose a risk to public health. Increased availability of these tests and subsidies to reduce the cost to the consumer may be appropriate. However, further studies investigating the incidence of these contaminants in Canadian water supplies, and their associations with health, should be explored.

Many respondents reported that it was important that they receive more information about water testing recommendations and options, test results from other private wells in their neighbourhoods, and advice on water treatment options. The City website and phone line, both of which require an active, information seeking behaviour on part of the residents, were the least likely media to be used by respondents in this study. A lack of internet access did not appear to be a cause for these results, as approximately 67% of households (161/239) reported having such access. Our results suggest that flyers would be the most effective route for delivery of this information in this population. Further, approximately half of the households reported being likely to use the newspaper, television and radio for this information if it was made available. The majority of people surveyed in two North American studies identified these media as the source of their drinking water information and only a small proportion identified medical doctors, health care professionals and the government as their sources [15,35]. It is important that the public receive credible and valid information. Given our results and the tendency for the public to use newspapers, television and radio as sources of information [15,35], the effectiveness of public health efforts may be improved by increasing the use of these media for information delivery.

The majority of wells in this study were constructed by drilling. Many wells however, were shallow and approximately 12% were dug wells, which tend to be more vulnerable to contamination [2,6,29]. Further, 10% and 16% of households did not know their well type or depth, respectively, suggesting unfamiliarity with their private water supply. With the exception of a higher number of "unknown" responses, these results are similar to another study performed in Southern Ontario [8]. We did not collect data on the state of repair or age of the wells, but older wells or those not well maintained are also at increased risk for contamination [2,29]. It may therefore be useful to highlight in dissemination efforts the importance of being familiar with one's well and the possible impact well type may have on water quality.

The nature of this study may have increased the potential for response bias. For ethical reasons, respondents were informed that the University of Guelph, the Public Health Agency of Canada (formerly Health Canada) and the City of Hamilton Health Protection Branch were conducting the study, which might have lead to increased responses towards health concerns. Further, the relatively low response rate (54%) may have contributed to selection bias. We did not have demographic information on non-respondents; hence, we could only compare our sample to the total City of Hamilton census population. Our respondents were older and reported slightly more members per household than the census population. We also had a lower proportion of people with formal education below grade nine and a higher proportion of high school and university graduates than in the census population. Finally, our sample had a smaller proportion of households in the lowest two household income brackets and a larger proportion in the highest income bracket. Therefore, while it may be reasonable to generalize the results of this study to similar communities in North America, there may exist limitations regarding the extent to which our results may be generalized.

## Conclusion

This study investigated the perceptions of drinking water held by residents with private water supplies in the City of Hamilton, Ontario. While respondents rated their water quality highly, the majority had concerns regarding the water from their private supply, and the use of bottled water and water treatment devices was extensive. Water testing was performed infrequently and for minimal parameters; hence, waterborne pathogens could go undetected and increase the public health risk for waterborne disease. Increased surveillance testing of private water supplies and investigation of their association with adverse health outcomes in this population is therefore warranted. Further, efforts to increase the testing of private water supplies in this population should be taken, including increased public education about the importance of regular water testing and changes to the testing process that would increase its convenience. Respondents clearly wanted more information on drinking water from private water supplies and reported being likely to use a number of specific media for such education. This understanding of the public's perceptions, concerns and self-identified needs will better enable the design and implementation of effective public health programs in this population. This will be integral to alleviating the public's concerns about drinking water and helping to create an informed, attentive and collaborative community.

## Competing interests

The author(s) declare that they have no competing interests.

## Authors' contributions

SEM and AQJ conceived the project; AQJ designed the study, administered the surveys, collected and analyzed the data, and drafted the manuscript; CED, KD, SEM, SAMc, DWT, EM and DJC and SJH provided critical feedback on the study design, questionnaire development, the analyses and interpretation of results, as well as editorial comments on the manuscript. DJC assisted with administration of the surveys. All authors read and approved the final manuscript.

## Pre-publication history

The pre-publication history for this paper can be accessed here:



## Figures and Tables

**Table 1:** Demographic comparison of survey respondents and residents of the City of Hamilton, Ontario

	**Census Population # (%)**	**Study Population # (%)**	**p-value (overall test)**
**Sex**			
Male	239 520 (49)	124 (52)	0.36
Female	250 750 (51)	115 (48)	
**Age (years)**			
20–29 *	62 650 (17)	9 (4)^†^	<0.0001
30–39	74 830 (21)	25 (11)^†^	
40–49	76 890 (21)	60 (25)	
50–59	57 185 (16)	63 (26)^†^	
60–69	40 135 (11)	50 (21)^†^	
70 and older	50 885 (14)	32 (13)	
**Education**			
Less than grade 9	36 915 (12)	12 (5)^†^	<0.0001
Grade 9–13, no high school diploma	70 465 (23)	44 (19)	
Grade 9–13, high school diploma	51 455 (16)	56 (24)^†^	
College or trade school diploma	101 395 (33)	71 (30)	
University, graduate or professional degree	50 930 (16)	53 (22)^†^	
**Total household income**			
<$10 000	12 575 (7)	2 (1)^†^	<0.0001
>$10 000 to <$20 000	25 240 (13)	8 (4)^†^	
>$20 000 to <$30 000	21 035 (11)	16 (8)	
>$30 000 to <$40 000	20 490 (11)	24 (13)	
>$40 000 to <$50 000	18 195 (10)	15 (8)	
>$50 000 to <$60 000	16 720 (9)	18 (10)	
>$60 000 to <$70 000	15 585 (8)	18 (9)^†^	
>$70 000	58 310 (31)	89 (47)	
**Mean number of people in household**	2.6	3.0	--

^† ^Count in that subcategory was significantly different (χ^2^>3.84, p < 0.05) between survey and census population

* Age range of comparison groups differ; census: 20–29 years versus sample: 18–29 years

**Table 2:** Respondent explanations for being "very concerned" or "concerned" about their private water supply safety^† ^(n = 123)

**Explanation**	**Frequency**	**Percent**
**Contamination**	62	41.6
(General)	(23)	(15.4)
(Pesticides/chemicals)	(20)	(13.4)
(Bacteria/fecal run-off)	(19)	(12.8)
**Disease/overall health**	38	25.5
**Fundamental necessity of water**	19	12.8
**Susceptibility of aquifer to external factors**	14	9.4
**Walkerton/media stories**	8	5.4
**Water testing**	6	4.0
(Distrust in testing/regulations)	(3)	(2.0)
(Minimal/no testing performed)	(2)	(1.3)
(Long test turnaround times)	(1)	(0.7)
**Lack of information on private water supplies**	2	1.3
**Total**	149*	100

^† ^as described in response to an open-ended question

* total number of explanations exceeds total number of respondents because of multiple explanations per respondent

**Table 3:** Respondents' concern level regarding potential contaminants/characteristics of water from their private water supplies

	**Very Concerned # (%)**	**Concerned # (%)**	**Neither Concerned nor Unconcerned # (%)**	**Unconcerned # (%)**	**Very Unconcerned # (%)**
Chemicals (n = 232)	127 (54.7)	50 (21.6)	21 (9.1)	24 (10.3)	10 (4.3)
Pesticides/fertilizers (n = 232)	129 (55.6)	56 (24.1)	21 (9.1)	17 (7.3)	9 (3.9)
Lead/other metals (n = 229)	115 (50.2)	58 (25.3)	26 (11.4)	21 (9.2)	9 (3.9)
Bacteria (n = 235)	146 (62.1)	66 (28.1)	9 (3.8)	10 (4.3)	4 (1.7)
Hardness (n = 232)	54 (23.3)	87 (37.5)	65 (28.0)	22 (9.5)	4 (1.7)
Smell (n = 230)	88 (38.3)	81 (35.2)	35 (15.2)	22 (9.6)	4 (1.7)

**Table 4:** Proportion of respondents for whom specific factors were important in deciding to use alternative water sources

**Factor**	**Very Important # (%)**	**Important # (%)**	**Total # (%)**
**Water treatment devices**			
Improved taste (n = 114)	53 (46.5)	38 (33.3)	91 (79.8)
Improved smell (n = 113)	52 (46.0)	35 (31.0)	87 (77.0)
Reduced bacteria (n = 115)	86 (74.8)	17 (14.8)	103 (89.6)
Reduced lead or other metals (n = 114)	67 (58.8)	26 (22.8)	94 (81.6)
Reduced chemicals (n = 114)	67 (58.8)	22 (19.3)	89 (78.1)
Reduced cloudiness (n = 113)	55 (48.7)	27 (23.9)	82 (72.6)
Reduced hardness (n = 114)	50 (43.9)	35 (30.7)	85 (74.6)
**Bottled water**			
Improved taste (n = 130)	59 (45.4)	31 (23.8)	90 (69.2)
Improved smell (n = 128)	47 (36.7)	36 (28.1)	83 (64.8)
Reduced bacteria (n = 131)	70 (53.4)	23 (17.6)	93 (71.0)
Reduced lead or other metals (n = 124)	54 (43.6)	22 (17.7)	76 (61.3)
Reduced chemicals (n = 127)	58 (45.7)	24 (18.9)	82 (64.6)
Reduced cloudiness (n = 125)	47 (37.6)	33 (26.4)	80 (64.0)
Reduced hardness (n = 124)	38 (30.7)	34 (27.4)	72 (58.1)
Better safety testing/control (n = 128)	64 (50.0)	23 (18.0)	87 (68.0)

**Table 5:** Proportion of respondents likely to use specific media for information on private water supplies

**Dissemination route**	**Very Likely # (%)**	**Likely # (%)**	**Total # (%)**
Radio (n = 218)	32 (14.7)	65 (29.8)	97 (44.5)
Television (n = 218)	43 (19.7)	75 (34.4)	118 (54.1)
Newspaper (n = 219)	52 (23.7)	87 (39.7)	139 (63.4)
Flyer/brochure (n = 227)	107 (47.1)	85 (37.4)	192 (84.5)
City of Hamilton website (n = 213)	22 (10.3)	35 (16.4)	57 (26.7)
City of Hamilton phone line (n = 204)	16 (7.8)	25 (12.3)	41 (20.1)

**Table 6:** Frequency with which 239 households in the City of Hamilton tested their private water supplies

**Frequency of testing**	**# Households**	**Percent**
Never	51	21.3
Less than once every 10 years	26	10.9
Once every 5–9 years	22	9.2
Once every 3–4 years	27	11.3
Once every 2 years	19	7.9
Once every year	54	22.6
Once every 6 months	21	8.8
Once every 3 months	9	3.8
More than 4 times a year	10	4.2

**Total**	239	100.0

**Table 7:** Respondent explanations for not testing, or not testing more frequently, their private water supplies ^†^

**Explanation**	**Frequency**	**Percent**
Inconvenience and time issues	39	26.5
No health problems or noticeable water changes	32	21.8
Don't drink from private water source	22	15.0
Forget or procrastinate	13	8.8
Lack of information on testing	10	6.8
No particular reason	10	6.8
Use a water treatment system	9	6.1
Previous test results were normal	8	5.4
Cost	3	2.0
Distrust laboratory	1	0.7

**Total**	147	100

^† ^as described in response to an open-ended question

**Table 8:** Proportion of respondents who report various methods would increase testing of private water supplies (n = 241)

**Proposed method to increase frequency of water testing among residents with private water supplies**	**Households # (%)**
Increased number of water sample drop-off locations	137 (56.9)
Reminder mail-outs (flyers/brochures)	120 (49.8)
Water collection bottles delivered to residences	119 (49.4)
Water samples picked up directly from residences	112 (46.5)
Reminders in newspaper advertisements	53 (22.0)
